# Intentions to Seek Mental Health Services During the COVID-19 Pandemic Among Chinese Pregnant Women With Probable Depression or Anxiety: Cross-sectional, Web-Based Survey Study

**DOI:** 10.2196/24162

**Published:** 2021-02-11

**Authors:** Qian Wang, Bo Song, Jiangli Di, Xue Yang, Anise Wu, Joseph Lau, Meiqi Xin, Linhong Wang, Phoenix Kit-Han Mo

**Affiliations:** 1 National Center for Women and Children's Health Chinese Centers for Disease Control and Prevention Beijing China; 2 Center for Health Behaviours Research School of Public Health and Primary Care The Chinese University of Hong Kong Hong Kong Hong Kong; 3 Department of Psychology Faculty of Social Sciences University of Macau Macau Macao; 4 National Center for Chronic and Noncommunicable Disease Control and Prevention Chinese Center for Disease Control and Prevention Beijing China

**Keywords:** pregnant women, COVID-19, depression, anxiety, help-seeking, mental health services, social support, trust, intention, mental health, pregnancy, survey

## Abstract

**Background:**

Mental health problems are prevalent among pregnant women, and it is expected that their mental health will worsen during the COVID-19 pandemic. Furthermore, the underutilization of mental health services among pregnant women has been widely documented.

**Objective:**

We aimed to identify factors that are associated with pregnant women’s intentions to seek mental health services. We specifically assessed pregnant women who were at risk of mental health problems in mainland China.

**Methods:**

A web-based survey was conducted from February to March, 2020 among 19,515 pregnant women who were recruited from maternal health care centers across various regions of China. A subsample of 6248 pregnant women with probable depression (ie, those with a score of ≥10 on the 9-item Patient Health Questionnaire) or anxiety (ie, those with a score of ≥5 on the 7-item General Anxiety Disorder Scale) was included in our analysis.

**Results:**

More than half (3292/6248, 52.7%) of the participants reported that they did not need mental health services. Furthermore, 28.3% (1770/6248) of participants felt that they needed mental health services, but had no intentions of seeking help, and only 19% (1186/6248) felt that they needed mental health services and had intentions of seek help. The results from our multivariate logistic regression analysis showed that age, education level, and gestational age were factors of not seeking help. However, COVID-19–related lockdowns in participants’ cities of residence, social support during the COVID-19 pandemic, and trust in health care providers were protective factors of participants’ intentions to seek help from mental health services.

**Conclusions:**

Interventions that promote seeking help for mental health problems among pregnant women should also promote social support from health care providers and trust between pregnant women and their care providers.

## Introduction

### Overview of the COVID-19 Pandemic

COVID-19 is an emerging infectious disease that has resulted in considerable public health risks across the globe. As of November 3, 2020, 91,921 COVID-19 cases and 4746 deaths have been reported in China, and 45,876,878 cases and 1,188,165 deaths have been reported outside of China [1]. The World Health Organization declared the COVID-19 epidemic a public health emergency of international concern on January 30, 2020. It was then declared a pandemic on March 11, 2020 [2]. The COVID-19 pandemic has also resulted in widespread fear, worry, and anxiety in the general public [3]. Documented evidence has shown that anxiety, depression, posttraumatic stress disorder, and psychological distress have been highly prevalent among the general public during the COVID-19 pandemic [4].

### Pregnant Women are at Risk of Mental Health Problems During the Pandemic

Mental disorders are the leading causes of disease burden among adult women [5]. Depression and anxiety are the most common mental disorders that occur during pregnancy; depression and anxiety affect 10%-30% of pregnant women [6,7]. During the COVID-19 pandemic, pregnant women may experience greater mental health burdens than the general population, as pregnant women face the challenges of both pregnancy and the epidemic. Pregnant women are more susceptible to SARS-CoV-2 infection due to their immunosuppressive state and their reduced tolerance to hypoxia, which are caused by pregnancy-induced physiological changes [8]. As such, pregnant women are an at-risk group that is in need of COVID-19 prevention and management services [9]. It is therefore expected that the severity of mental disorders among pregnant women will increase during the COVID-19 pandemic. Empirical studies have documented a high global prevalence of depression (ie, 5.2%-40%) and anxiety (ie, 3.8%-72%) among pregnant women during the COVID-19 pandemic.

Prenatal mental disorders not only impair social, cognitive, and psychological functions, but also lead to numerous adverse outcomes in both mothers and children, such as a high risk of preterm birth, intrauterine growth restrictions, maternal depressive disorder after birth, poor mother-child interactions, and poor child psychopathology and development [10-14]. Mental disorders also increase the risk of infection, including the risk of SARS-CoV-2 infection. This may be due to cognitive impairments, low risk perceptions, poor compliance with personal protection measures, and confined conditions in psychiatric wards [15].

### The Low Prevalence of Seeking Psychological Help Among Pregnant Women With Mental Problems

Despite the high prevalence and detrimental consequences of mental disorders, most people who exhibit mental disorder symptoms do not seek professional help and proper treatment [16,17]. It has been estimated that only 30% of individuals with mental illnesses across the globe have received treatment [16,17]. Studies have found that only 13.6%-33.3% of women with perinatal depression or anxiety have sought help from a health care professional [18-20]. This poor help-seeking pattern is even more pronounced in Chinese women [21]. In China, an intense level of stigma is attached to mental illness. The ongoing COVID-19 pandemic can further reduce the use of mental health services [22,23]. Identifying factors of seeking help for mental disorders during an epidemic period is important in guiding public education efforts that promote help-seeking and alleviate the barriers that prevent pregnant women from seeking mental health services.

### Factors Associated With Seeking Mental Health Services

Several studies have identified a number of facilitators and barriers with regard to seeking mental health services among perinatal women. Background factors such as young age [18,20], low levels of education [24], and first-time births [24] have been associated with a low likelihood of seeking mental health services among perinatal women. Alternatively, a history of psychiatric or psychological treatment [18], increased symptom severity, and long disease durations have been associated with a high likelihood of seeking mental health services. Several studies have also found that favorable attitudes toward psychiatric and psychological treatment are facilitators of seeking help from mental health services [7,18]. However, poor knowledge about mental symptoms, the inability to recognize mental symptoms [25-27], a lack of knowledge about available psychosocial services and their benefits [20,28], the stigma associated with perinatal mental illness [7,29,30], a fear of being labelled as mentally ill [25,28], the inability to self-disclose information due to feelings of embarrassment [25,26], and a tendency to conceal distressing and negative personal information [27,30] are common hindrances to seeking mental health services among perinatal women. Practical or structural barriers such as concerns of monetary costs, work constraints, childcare responsibilities, and the limited accessibility of mental health service are also common hinderances to seeking help from mental health services among perinatal women [7,19,20,25,28].

### The Roles of Social Support and Trust in Care Providers in Seeking Mental Health Services

Despite the wealth of factors identified in literature, relatively few studies have assessed the roles of interpersonal factors in Chinese pregnant women’s intentions to seek mental health services. Theoretically, psychological reactions at both the individual and societal level are relevant to managing behaviors and improving emergency preparedness during pandemics [31]. Several studies have reported that significant others have a positive influence on people’s intentions to seek help from professionals [32,33]. Studies among perinatal women have revealed that perceived encouragement from their partners can predict perinatal women’s intentions to seek formal help [34], and mediate the association between perinatal women’s intentions to seek informal help and their intentions to seek professional help [35]. Studies among women with postpartum depression have also reported that a lack of social support is a significant barrier to seeking mental health services [25,26,28]. It has been contended that behavioral intention is more subject to influences from significant others when a behavior is not well incorporated into one’s repertoire [36]. In terms of the performance of a behavior, a lack of personal experience can lead an individual to further rely on their significant others’ views and encouragements [37]. We believed that the participants in our study would value the influence that social support has on their decisions to seek mental health services during the COVID-19 period.

Health professionals’ positive influence on the promotion of help-seeking behaviors has been documented in literature. A meta-synthesis of factors that affect women’s decisions to seek help for perinatal distress has identified the influence of health care professionals as one of the major factors of help-seeking [30]. Trust and familiarity with care providers may play an even more important role than perceived need in terms of shaping help-seeking intentions and behaviors [38]. Several mothers have also listed empathy and kindness as key attributes that ideal health professionals should have when providing mental health services [28]. Alternatively, disappointment and a lack of trust in care providers have been cited as barriers to seeking mental health services among pregnant women [27,39].

It has been contended that the importance of social support and trust in care providers in psychological help-seeking is evident in China. It has long been documented that the Chinese culture values interpersonal relatedness, which encompasses harmony with others and the consideration of the self, in terms of family and community relationships [40,41]. Additionally, being obedient to authority figures is emphasized in Chinese culture [42]. Pregnant women may be obligated to seek and receive professional help for mental problems when they think that their significant others and care providers will support them in doing so.

### Research Gaps and Study Objectives

Despite the wealth of literature on help-seeking among perinatal women with mental disorders, little is known about help-seeking intentions among Chinese pregnant women with probable depression or anxiety during the COVID-19 pandemic. This study aimed to examine intentions to seek mental health services among pregnant women who are at risk of mental health problems during the COVID-19 period in China, and to identify the background, pregnancy-related, contextual, and interpersonal factors that are associated with help-seeking intentions.

## Methods

### Inclusion and Exclusion Criteria

In this study, a subsample of pregnant women who were at risk of depression and anxiety were identified from a whole sample of pregnant women. The inclusion criteria for the whole sample were as follows: (1) female sex, (2) age of ≥18 years, (3) current pregnancy, and (4) the intention to continue the pregnancy. Pregnant women who planned to terminate their pregnancy were excluded from this study.

### Procedure

A web-based, cross-sectional survey was conducted from February 24 to March 10, 2020. Eligible participants were identified from the records of maternal health care centers from various regions of China, and invited to take part in the web-based survey through a phone app (ie, WeChat [Tencent Inc]). Interested participants visited the web-based survey through a link or quick response code and provided informed consent before starting the survey. Participants were assured that the study was anonymous and confidential, and that declining to take part in the survey would not affect the services they obtained. The survey took 15-20 minutes to complete. Ethical approval was obtained from the authors’ institution.

A total of 19,515 completed responses were collected from the web-based survey. Our subsample consisted of pregnant women who were at risk of mental health problems, which were defined as scores that exceeded the cutoffs for probable depression and probable anxiety. Of the 19,515 participants, 6248 (32%) participants fulfilled the criteria (both probable depression and probable anxiety: 2595/19,515, 13.3%; only probable depression: 552/19,515, 2.8%; only probable anxiety: 3101/19,515, 15.9%) and were included in the analysis. The levels of depression and anxiety among the 19,515 participants are shown in Multimedia Appendix 1.

### Measures

#### Sociodemographic and Pregnancy-Related Characteristics

Participants were asked to report their age, education level, parity, gestational age, and whether they had any pregnancy-related complications.

#### Isolation Due to COVID-19

Participants were asked to report whether their city of residence was locked down, and whether they were being quarantined due to COVID-19.

#### Social Support

Participants were asked to rate the level of social support that they received during the COVID-19 pandemic (eg, support from family, friends, or significant others) on a 10-point Likert scale (ie, 1=very little, 10=very much). Similar items have been used in previous studies [43].

#### Trust in Care Providers

Trust in care providers was measured with the affect- and cognition-based trust (ACT) scale, which was developed by McAllister [44]. The ACT scale consists of 8 items, of which 4 assess cognition-based trust and 4 assess affect-based trust. Items are rated on a 5-point scale (ie, 1=strongly disagree, 5=strongly agree). Higher scores indicate higher levels of trust in care providers. The internal reliability of the scale was satisfactory in this study (Cronbach α=.95).

#### Intention to Seek Mental Health Services

Participants were asked to rate their intentions to seek mental health services by choosing 1 of the following 3 options: option 1, which stated “I don’t need mental health services”; option 2, which stated “I need mental health services, but I will not seek help from these services”; and option 3, which stated “I need mental health services and I will seek help from these services.” Those who chose option 3 were considered to have intentions to seek mental health services.

The following two measures were used to screen those participants for probable depression or anxiety: 9-item Patient Health Questionnaire (PHQ-9) scores and 7-item Generalized Anxiety Disorder Scale (GAD-7) scores.

Probable depression was measured by using the Chinese version of the PHQ-9 [45], which has been widely used in the Chinese population [46,47]. Participants were asked to rate how often they were bothered by the symptoms in the 2 weeks before taking the survey, on a 4-point Likert scale (ie, 0=not at all, 3=almost every day). The total score can range from 0 to 27. Higher scores indicate higher levels of depression. Those with a score of ≥10 were considered to have probable depression [48].

Probable anxiety was assessed by using the Chinese version of the GAD-7 [49], which has been used in the Chinese population [50,51]. Participants were asked to rate how often they were bothered by each symptom in the 2 weeks before taking the survey, on a 4-point Likert scale (ie, 0=not at all, 3=nearly every day). The total GAD-7 score can range from 0 to 21. Higher scores indicate higher levels of anxiety. Those with a score of ≥5 were considered to have probable anxiety [49].

### Statistical Analysis

We provided descriptive statistics. A Spearman correlation analysis was conducted to examine the relationships between social support and trust in care providers and sociodemographic/pregnancy-related characteristics. Univariate logistic regression analyses were conducted to examine the association between independent variables and intentions to seek mental health services, and respective univariate odds ratios (ORus) and 95% confidence intervals were calculated. To produce a final model that accounted for all independent variables, a multivariate logistic regression analysis was conducted with all independent variables by using the forward enter method, which allowed us to calculate multivariate odds ratios (ORms). Data analyses were performed with SPSS version 21.0 (IBM Corp), and a *P* value of <.05 was considered statistically significant.

## Results

### Descriptive Statistics of the Participants

Slightly less than two-thirds (3984/6248, 63.8%) of participants were aged ≤30 years. A similar number of participants (3681/6248, 59%) had a postsecondary level of education. More than half (3594/6248, 57.5%) of the participants were nulliparous, and around half (3146/6248, 50.4%) were in their third trimester. A small number (598/6248, 9.6%) of participants reported that they experienced pregnancy-related complications. About two-thirds (4065/6248, 65.1%) of participants reported that their city of residence was locked down due to COVID-19, and a small number (311/6248, 5%) of participants were quarantined due to COVID-19 at the time of this study (Table 1).

**Table 1:** Descriptive statistics of the participants (N=6248).

Sociodemographic characteristics			Value, n (%)
**Age (years)**			
	≤19		107 (1.7)
	20-25		1229 (19.7)
	26-30		2648 (42.4)
	31-35		1756 (28.1)
	36-40		435 (7)
	≥41		73 (1.2)
**Education level**			
	Primary or below		134 (2.1)
	Junior secondary		1168 (18.7)
	Senior secondary		1265 (20.2)
	Matriculation		1635 (26.2)
	Undergraduate		1703 (27.3)
	Postgraduate or above		343 (5.5)
**Pregnancy-related characteristics**			
	**Parity**		
		Nulliparous	3594 (57.5)
		Primiparous	2347 (37.6)
		Multiparous	307 (4.9)
	**Gestational age (weeks of pregnancy)**		
		First trimester (≤12)	808 (12.9)
		Second trimester (13-26)	2294 (36.7)
		Third trimester (≥27)	3146 (50.4)
	**Pregnancy-related complications**		
		No	5650 (90.4)
		Yes	598 (9.6)
**Isolation due to COVID-19**			
	City of residence locked down due to COVID-19		4065 (65.1)
	Quarantined due to COVID-19		311 (5)
**Intention to seek mental health services**			
	I don’t need mental health services		3292 (52.7)
	I need mental health services, but I will not seek help from these services		1770 (28.3)
	I need mental health services and I will seek help from these services		1186 (19)

### Social Support, Trust in Care Providers, and Intentions to Seek Mental Health Services

In the subsample of participants who had probable depression and probable anxiety, the mean score for social support that participants received during the COVID-19 pandemic was 8.06 (SD 2.28). The mean score for trust in care providers was 33.02 (SD 5.34). More than half of the participants (3292/6248, 52.7%) felt that they did not need mental health services, and about a quarter (1770/6248, 28.3%) felt that they needed mental health services, but would not seek help from these services. Only 19% (1186/6248) of participants stated that they needed mental health services and had the intention to seek help from these services.

### Correlations Between Social Support and Trust in Care Providers and Sociodemographic/Pregnancy-Related Characteristics

Social support that participants received during the COVID-19 period significantly correlated with older age (*r*_s_=.03; *P*=.007) and high parity (*r*_s_=.05, *P*<.001). Social support did not significantly correlate with education level (*r*_s_=.003, *P*=.79), the presence of pregnancy-related complications (*r*_s_=−.01, *P*=.70), or gestational age (*r*_s_=−.01, *P*=.38). Trust in care providers significantly correlated with high parity (*r*_s_=.03, *P*=.03), but not with age (*r*_s_=.01, *P*=.43), education level (*r*_s_=.01, *P*=.56), gestational age (*r*_s_=−.01, *P*=.30), or the presence of pregnancy-related complications (*r*_s_=.02, *P*=.25).

### Regression Models for Intentions to Seek Help From Mental Health Services

The results from the univariate logistic regression analyses (Table 2) showed that among the background characteristics, older age (ORus ranged from 0.43 to 0.60), high levels of education (ORus ranged from 0.43 to 0.68), and high gestational age (ORus ranged from 0.65 to 0.69) were associated with having no intention to seek mental health services, while multiparity (ORu 1.45, 95% CI 1.10-1.91) was associated with having the intention to seek mental health services. Among the isolation-related factors, COVID-19–related lockdowns in participants’ cities of residence (ORu 1.26, 95% CI 1.10-1.44) were associated with having the intention to seek mental health services. Among the interpersonal factors, both trust in health care providers (ORu 1.04, 95% CI 1.02-1.05) and social support that participants received during the COVID-19 pandemic (ORu 1.07, 95% CI-1.04-1.10) were associated with having the intention to seek mental health services.

The results of the multivariate logistic regression (Table 2) were similar to those of the univariate logistic regressions. Among the background characteristics, older age (ORms ranged from 0.49 to 0.67), high levels of education (ORms ranged from 0.47 to 0.68), and high gestational age (ORms ranged from 0.66 to 0.70) were associated with low intentions to seek mental health services. Among the isolation-related factors, COVID-19–related lockdowns in participants’ cities of residence (ORm 1.17, 95% CI 1.01-1.35) were associated with having the intention to seek mental health services. Among the interpersonal factors, both trust in health care providers (ORm 1.03, 95% CI 1.02-1.04) and social support that participants received during the COVID-19 period (ORm 1.05, 95% CI 1.02-1.08) were associated with having the intention to seek mental health services.

**Table 2:** Logistic regression analysis of intentions to seek mental health services among pregnant women with probable depression and anxiety (N=6248).

Sociodemographic characteristics			Intention to seek mental health services			
			ORu^a^ (95% CI)	*P* value	ORm^b^ (95% CI)	*P* value
**Age (years)**						
	≤19		1 (referent)	N/A^c^	1 (referent)	N/A
	20-25		0.60 (0.39-0.92)	.02^d^	0.67 (0.44-1.04)	.08
	26-30		0.43 (0.28-0.64)	<.001^e^	0.50 (0.32-0.77)	.002^f^
	31-35		0.46 (0.30-0.71)	<.001^e^	0.53 (0.34-0.82)	.005^f^
	36-40		0.47 (0.29-0.75)	.002^f^	0.50 (0.30-0.83)	.008^f^
	≥41		0.49 (0.24-0.99)	.047^d^	0.49 (0.23-1.01)	.05
**Education level**						
	Primary or below		1 (referent)	N/A	1 (referent)	N/A
	Junior secondary		0.68 (0.46-1.01)	.06	0.68 (0.46-1.01)	.06
	Senior secondary		0.52 (0.35-0.76)	<.001^e^	0.55 (0.37-0.83)	.004^f^
	Matriculation		0.43 (0.29-0.63)	<.001^e^	0.47 (0.32-0.71)	<.001^e^
	Undergraduate		0.46 (0.31-0.67)	<.001^e^	0.53 0(.36-0.80)	002^f^
	Postgraduate or above		0.52 (0.33-0.82)	.005^f^	0.66 (0.41-1.06)	.08
**Pregnancy-related characteristics**						
	**Parity**					
		Nulliparous	1 (referent)	N/A	1 (referent)	N/A
		Primiparous	1.04 (0.91-1.18)	.61	1.02 (0.88-1.19)	.78
		Multiparous	1.45 (1.10-1.91)	.007^f^	1.24 (0.92-1.68)	.16
	**Gestational age (weeks of pregnancy)**					
		First trimester (≤12)	1 (referent)	N/A	1 (referent)	N/A
		Second trimester (13-26)	0.65 (0.53-0.78)	<.001^e^	0.66 (0.55-0.81)	<.001^e^
		Third trimester (≥27)	0.69 (0.57-0.84)	<.001^e^	0.70 (0.58-0.84)	<.001^e^
	**Pregnancy-related complications**					
		No	1 (referent)	N/A	1 (referent)	N/A
		Yes	1.02 (0.82-1.26)	.87	1.12 (0.90-1.40)	.31
**Isolation due to COVID-19**						
	**City of residence locked down due to COVID-19**					
		No	1 (referent)	N/A	1 (referent)	N/A
		Yes	1.26 (1.10-1.44)	.001^f^	1.17 (1.01-1.35)	.03^d^
	**Quarantined due to COVID-19**					
		No	1 (referent)	N/A	1 (referent)	N/A
		Yes	1.28 (0.97-1.68)	.08	1.19 (0.90-1.56)	.23
**Interpersonal factors**						
		Trust in health care provider	1.04 (1.02-1.05)	<.001^e^	1.03 (1.02-1.04)	<.001^e^
		Received social support during the COVID-19 period	1.07 (1.04-1.10)	<.001^e^	1.05 (1.02-1.08)	.001^f^

^a^ORu: univariate odds ratio; the odds ratio derived from the univariate logistic regression analysis.

^b^ORm: multivariate odds ratio; the odds ratio derived from multivariate logistic regression analysis. This analysis was conducted by using the forward enter method.

^c^N/A: not applicable.

^d^Statistically significant at a level of *P*<.05.

^e^Statistically significant at a level of *P*<.001.

^f^Statistically significant at a level of *P*<.01.

## Discussion

### Principal Findings

The COVID-19 pandemic has resulted in considerable distress and has potentially worsened the mental health of the public [3]. Pregnant women are one of the at-risk groups that may have elevated mental risks. It is important to understand the factors of help-seeking intentions among pregnant women with mental health risks during an epidemic period, as early intervention can reduce the negative impact that mental problems can have on both mothers and children in the long term. It is important to note that among our sample of pregnant women who were at risk of mental problems, less than half (2956/6248, 47.3%) of the participants felt the need to seek mental health services. Our findings are in line with those of several studies that have documented a low level of perceived need for mental health services [52,53]. This low level of perceived need could be explained by low levels of knowledge and awareness with regard to the importance of mental health problems [54,55]. Studies on perinatal Chinese American women have revealed that several women believe that postpartum depression does not exist in China [56]. Furthermore, the somatization of mental illness has been commonly reported in China [57]. If pregnant women’s psychological distress appeared in the form of somatic symptoms, they may be more likely to seek medical help. Pregnant women might also tend to view a disturbed mood as a normal part of pregnancy [27,56], thereby resulting in the underreporting of the need to seek help.

Despite having mental symptoms, it is surprising that only 19% of our sample reported that they needed and intended to seek mental health services. Our findings were in line with the underutilization of mental health services, which has been documented across several countries and populations [17,53,58,59]. Negative perceptions toward mental health services have been frequently documented among pregnant women. Studies have found that perinatal women often view psychological treatment as ineffective, and describe mental health providers as uncaring, impersonal, and emotionally detached [27,60]. The low levels of psychological help-seeking intentions can also be explained by the incompatibility between Chinese culture and the general public’s views on mental illness. For example, in Chinese culture, psychological distress and mental illness are often viewed as a lack of self-control in solving one’s own problems, personal weaknesses, bad thoughts, or a lack of will power [56]. Such negative perceptions of mental illness might result in the intense stigmatization and discrimination of people with mental illnesses [61]. Being dismissive of mental health problems and having concerns about feeling embarrassed when seeking help might also reduce people’s intentions to seek mental health services when they are faced with mental problems [61]. Furthermore, due to the influence of traditional Chinese culture, the participants in this study might have preferred to seek help from traditional/indigenous healers or spiritual outlets for alleviating their mental symptoms, rather than seek help from mental health professionals [62].

This study identified a number of background factors that were associated with seeking mental health services. In contrast to studies that have stated that older age was associated with high levels of help-seeking among perinatal women [18,20,63], our study revealed that older perinatal women had low intentions to seek mental health services. Older pregnant women are at a high risk of pregnancy-related complications. Therefore, they might be more attentive to their pregnancy-related needs than their own mental needs. Furthermore, studies have shown that older individuals exhibit negative attitudes toward seeking mental services and are more sensitive to stigmas that are associated with mental illness [64,65]. Older women might avoid seeking help from mental health professionals to minimize the feelings of shame that come from seeking help.

In contrast to studies that have stated that high levels of education are associated with high levels of psychological help-seeking in perinatal women [18,63], our study showed that pregnant women with high levels of education tended to report that they had no intentions to seek mental health services. It is plausible that women who are more educated than others are more likely to attribute their mental symptoms to other causes (eg, work stress) or believe that they can handle mental health challenges on their own [66], thereby reducing their intentions to seek mental health services. It has been suggested that perinatal women with higher education levels have more difficulties in recognizing the presence of psychological problems than those with lower education levels [18]. These difficulties act as considerable barriers to help-seeking.

Among the pregnancy-related characteristics that were assessed in this study, high gestational age was associated with low intentions to seek mental health services. Compared to pregnant women with low gestational ages, those with high gestational ages might be more likely to believe that increased stress and distress are normal as the time to delivery becomes shorter.

The effects of interpersonal factors on help-seeking are indispensable. In this study, participants who received high levels of social support during the COVID-19 pandemic were more likely to seek psychological help than those who received low levels of social support. Our findings corroborate those of existing studies that report significant associations between social support and positive attitudes toward seeking mental health services [67,68], social support and the perceived need for psychological help [69], and social support and psychological help-seeking intentions and behaviors [67,70,71]. The effects of social support on help-seeking intention may be more important in a collectivistic culture that respects normative values. The level of people’s intentions to seek help for mental problems is likely to increase when they receive support from significant others, especially during the COVID-19 pandemic.

Several studies have emphasized that health care providers play a role in facilitating the process of professional help-seeking. Our study demonstrates that pregnant women who had high levels of trust in care providers were more likely to report an intention to seek mental health services than those with low levels of trust in care providers. This finding confirms that perceiving health care providers as helpful and trustworthy people is an important factor of help-seeking behaviors [27,28]. Compared to individuals who have a low level of trust in health care providers, those who have a level of trust in care providers might be more likely to believe that their health care providers are dependable and view care providers’ advice and services as helpful and effective. They may also be more likely to experience higher levels of comfort when expressing their negative thoughts to people who they believe are caring and trustworthy [27]. Our findings further support the belief that trust in care providers plays an important role in facilitating help-seeking intentions among people in collectivistic societies, wherein interpersonal relatedness and strong kinship bonds are particularly emphasized.

### Implications

Our study has important implications for increasing the acceptance and use of mental health services among Chinese pregnant women. The low perceived needs and intentions to seek mental health services that were documented in this study emphasize the need to increase people’s awareness of perinatal mental disorders, and promote positive attitudes toward psychological help-seeking among pregnant women. Mental health education has been found to be effective in improving knowledge, attitudes, and behaviors that are associated with mental health [72]. Mental health education for pregnant women should provide them with methods for identifying, understanding, and responding to signs of mental symptoms. Increasing pregnant women’s understanding of distress triggers during pregnancy will also promote positive attitudes toward perinatal mental disorders and increase their willingness to seek help. Such education should also be specifically adapted to perinatal mental disorders, and delivered through community or health care settings.

Our study also indicates that significant others who provide support to pregnant women who experience mental health challenges increase help-seeking intentions among pregnant women. The significant effects of social support in this study suggest that significant others, such as partners, family members, and close friends, should put more effort into promoting health-seeking intentions. Health professionals should also encourage discussions (ie, discussions about emotional problems and concerns) between significant others and pregnant women. This not only strengthens pregnant women’s perceived level of support, but also promotes the disclosure of personal issues, which is an essential first step in seeking mental health services. Significant others should also be included in mental health promotion programs for pregnant women.

The findings of our study also suggest that women are more likely to discuss their concerns with, and seek help from, mental health professionals if they believe that health care workers trustworthy. Empathetic communication and relationships between patients and care providers, and customized patient-centered care promote people’s trust in care providers and increase the use of mental health services [73]. Health care professionals should receive training on providing quality services for pregnant women. Such training should emphasize attitude, respect, and sensitivity toward the specific needs of pregnant women. Health care professionals should also receive training on interpersonal skills that help establish rapport and promote communication in clinical settings.

### Limitations

Our study has several limitations that should be noted. First, this study was cross-sectional in nature. Therefore, causality among the variables cannot be assumed. Second, we only measured probable depression and anxiety, as clinical diagnoses of depression and anxiety cannot be obtained from web-based surveys. Third, due to the cross-sectional design of this study, information on actual help-seeking behaviors was not obtained, as it seemed inappropriate to assess the association between independent variables and past help-seeking behaviors. Fourth, due to concerns about the length of the questionnaire, social support was only measured with a single survey item. Therefore, our survey results do not show the specific sources (eg, family or friends) and nature (eg, emotional or instrumental support) of the social support that participants received during the pandemic. Similarly, the ACT scale does not capture certain aspects of people’s trust in care providers, such as specific reasons for people’s trust and the nature of people’s trust (eg, fidelity, competence, and honesty) [74]. Hence, the potentially disproportionate effects of different types of social support and people’s trust in care providers could not be assessed in this study. Fifth, this study only assessed participants’ intentions to seek help from mental health services. Participants’ intentions to seek help from other sources were not assessed in this study. Future studies should be longitudinal in design and identify participants with depression and anxiety disorders by using validated diagnostic tools.

### Conclusion

This study shows that the perceived needs and intentions to seek mental health services during the COVID-19 period were low among Chinese pregnant women who were at risk of mental health problems. Social support during the COVID-19 pandemic and trust in care providers were protective factors of help-seeking intentions. There is an urgent need to improve perceived social support and promote trusting relationships between pregnant women and their care providers. These factors increase pregnant women’s intentions to seek help for mental health problems.

## Appendix

Multimedia Appendix 1 Levels of depression and anxiety among the total sample of 19,515 participants.

## Abbreviations

ACT: affect- and cognition-based trust
GAD-7: 7-item Generalized Anxiety Disorder Scale
ORm: multivariate odds ratio
ORu: univariate odds ratio
PHQ-9: 9-item Patient Health Questionnaire
