# Supplementary material for: Intentions to Seek Mental Health Services During the COVID-19 Pandemic Among Chinese Pregnant Women With Probable Depression or Anxiety: Cross-sectional, Web-Based Survey Study
Source: JMIR Ment Health. 2021 Feb 11;8(2):e24162. doi: 10.2196/24162 (PMC7879730; doi:10.2196/24162)
Supplement: Multimedia Appendix 1 [file mental_v8i2e24162_app1.docx]

Multimedia Appendix 1. Levels of depression and anxiety among the total sample of 19,515 participants.

| Levels | n (%) |
| --- | --- |
| **Depression measured by the 9-item Patient Health Questionnaire** | |
| Scored 0-4 | 10,803 (55.4) |
| Scored 5-9 | 5,565 (28.5) |
| Scored 10-14 | 2,053 (10.5) |
| Scored 15-19 | 793 (4.1) |
| Scored 20-27 | 301 (1.5) |
| Mean ± standard deviation | 5.00 ± 5.02 |
| **Anxiety measured by the 7-item General Anxiety Disorder Scale** | |
| Scored 0-4 | 13,819 (70.8) |
| Scored 5-9 | 4,177 (21.4) |
| Scored 10-14 | 1,052 (5.4) |
| Scored 15-21 | 467 (2.4) |
| Mean ± standard deviation | 3.19 ± 4.21 |
